# Diagnostic Value of Diurnal Variability of Orthostatic Heart Rate Increment in Children and Adolescents With POTS

**DOI:** 10.3389/fped.2021.644461

**Published:** 2021-05-13

**Authors:** Hong Cai, Shuo Wang, Runmei Zou, Fang Li, Juan Zhang, Yuwen Wang, Yi Xu, Cheng Wang

**Affiliations:** ^1^Department of Pediatric Cardiovasology, Children's Medical Center, The Second Xiangya Hospital, Central South University, Changsha, China; ^2^Jishou University School of Medicine, Jishou, China

**Keywords:** postural tachycardia syndrome, heart rate increment, diurnal variability, diagnosis, children, adolescents

## Abstract

**Objectives:** This study aims to investigate the diurnal variability of heart rate (HR) increment after standing (ΔHR) in pediatric postural tachycardia syndrome (POTS) and explore appropriate cutoff values of ΔHR at different times for the POTS diagnosis.

**Materials and Methods:** Seventy-eight patients (9–14 years) who presented with orthostatic intolerance symptoms were enrolled. Forty-three patients were diagnosed as POTS (ΔHR ≥40 bpm), and 35 patients were assigned to the non-POTS group (ΔHR <40 bpm). Twenty-six healthy children served as the control group. All subjects completed three standing tests in the morning, afternoon, and evening. Orthostatic HR parameters were analyzed to predict the diagnosis of POTS. Additionally, 41 patients were recruited as an external validation group.

**Results:** Orthostatic HR increments in both the POTS and non-POTS groups exhibited diurnal variability, which was markedly larger in the morning (*P* < 0.05), whereas it did not differ with the time of day in the control group. Among the POTS patients, 100% met the diagnostic criteria for POTS in the morning, 44.2% in the afternoon, and 27.9% in the evening. Almost half of the POTS patients (51.2%) displayed a positive result only in the morning standing test. However, in the three standing tests at different times, ΔHR from 1 to 10 min after standing and ΔHR_max_ were the highest in the POTS group compared with in the non-POTS and control groups (*P* < 0.05). Furthermore, the maximum ΔHR (ΔHR_max_) and ΔHR at 5 and 10 min in the afternoon and evening standing tests yielded moderate predictive values for the POTS diagnosis. The external validation test showed that the afternoon ΔHR_max_ ≥30 bpm to diagnose POTS yielded sensitivity, specificity, and accuracy of 85, 71.4, and 78%, respectively, and the evening ΔHR_max_ ≥25 bpm yielded sensitivity, specificity, and accuracy of 85, 76.2, and 80.5%, respectively.

**Conclusions:** The orthostatic HR increment exhibits diurnal variability in children and adolescents with POTS that may affect the diagnosis of POTS. Supplementary criteria are proposed for the POTS diagnosis based on diurnal variability.

## Introduction

Postural tachycardia syndrome (POTS) is a common form of pediatric orthostatic intolerance (OI), characterized by an excessive heart rate (HR) increment after standing and chronic day-to-day OI symptoms, such as dizziness, headache, palpitations, chest discomfort, blurred vision, profuse perspiration, and even syncope (1). Autonomic dysfunction is the principal mechanism for POTS. Many POTS patients have high plasma norepinephrine levels and excessive sympathetic activation upon standing (2, 3). In humans, almost every physiological system has some degree of the circadian rhythm. Many cardiovascular parameters, such as HR, blood pressure (BP), plasma catecholamine concentrations, and sympathetic activity, show distinct circadian rhythm, increasing significantly during the day and decreasing during the night (4). Various cardiovascular events, such as arrhythmias, angina pain, myocardial infarction, stroke, sudden cardiac death, and syncope, exhibit diurnal patterns with morning peaks (5, 6). Similarly, the diurnal variability in POTS adults is observed with orthostatic HR increment shown larger in the morning than in the afternoon or evening (7, 8). Some patients who meet the criteria for POTS in the morning do not always meet the criteria in the afternoon or the evening.

However, there was a lack of studies at present on the diurnal variability of pediatric POTS. Cardiovascular function and the diurnal variability of cardiovascular function are affected by aging (9, 10). We hypothesized that diurnal variability of orthostatic HR increments also existed in children and adolescents with POTS. If so, the existing HR criteria for pediatric POTS diagnosis may not always be appropriate for the standing test or head-up tilt test (HUTT) at different times of the day.

The key diagnostic criterion for POTS in children and adolescents is an HR increment of ≥40 bpm within the initial 10 min of the standing test or HUTT in the absence of orthostatic hypotension (11), but these are not universally accepted. Boris et al. (12) reported that a 40-bpm threshold for children and adolescents on the standing test may be too high because of no significant difference of symptomatic burden between OI pediatric patients with HR increments of 30–39 and ≥40 bpm. We sought to ascertain whether there was a difference in diurnal variability of orthostatic HR increment between OI patients with HR increments of <40 and ≥40 bpm.

Therefore, in the present study, we aimed to investigate the diurnal variability of HR increment after standing in children and adolescents with POTS and explore appropriate cutoff values of HR increment at different times for the POTS diagnosis. We also intended to evaluate the difference in diurnal variability of HR increment between patients with OI symptoms meeting and not meeting the HR criterion of a 40-bpm threshold.

## Participants and Methods

### Participants

Seventy-eight patients (9–14 years) who presented with OI symptoms were admitted to the Children's Medical Center, The Second Xiangya Hospital, Central South University from 2019 to 2020. The main causes of admission were recurrent dizziness, headache, palpitations, chest discomfort, and syncope. All patients received both HUTT in the morning and standing tests at three different time points within 3 days. Among them, 43 patients who met the HR criterion of a 40-bpm threshold both on the HUTT and on at least one standing test were recruited for the ≥40 bpm group (POTS group), and the remaining 35 patients who did not meet the HR criterion of a 40-bpm threshold were recruited for the <40 bpm group (non-POTS group). Twenty-six healthy children were recruited as the control group who had no history of OI symptoms and normal findings in physical examination, 12-lead electrocardiogram (ECG), and standing test. No one in the control group met the HR criterion of POTS. Cardiovascular, neurological, metabolic, and autoimmune diseases and others (such as infections, arrhythmia, severe anemia, recent long-term bed rest, or deconditioning) that could cause OI symptoms were excluded by a standard basic evaluation that consisted of careful history, physical examination, routine laboratory tests (complete blood count, blood biochemistry, thyroid function, 25-hydroxyvitamin D, autoimmune antibodies), ECG, 24-h Holter ECG, 24-h ambulatory BP monitor, echocardiogram, electroencephalography, and cranium CT/MRI.

Another 41 patients with frequent OI symptoms who were admitted to our hospital in 2020 were recruited for the external validation study. Among them, 20 patients were diagnosed as POTS with an HR increment of ≥40 bpm, and 21 patients were classified as the non-POTS group based on clinical OI symptoms and the morning standing test. The research was approved by the Medical Ethical Committee, The Second Xiangya Hospital, Central South University. All parents or guardians of the subjects signed informed consent.

### HUTT and Standing Test

All drugs that might affect the autonomic nervous function were avoided for at least five half-lives before evaluation. The test environment requires a quiet, dim, and suitable temperature. For the HUTT, the tilting device was the SHUT-100 tilt test monitoring software system of Beijing Standley Technology Co., Ltd. (Beijing, China). The subjects were kept supine on the tilt bed over 10 min, and their HR, BP, and ECG were monitored and recorded. After HR and BP stabilized, the bed was tilted upward to a 60° angle within 15 s, and HR, BP, and ECG were continuously monitored over 10 min. HUTT was conducted from 8:00 to 11:00 AM with subjects in a fasting state. On a different day, but within 3 days, the subjects performed standing tests in the morning (between 7:00 and 9:00 AM) in a fasting state, in the afternoon (between 3:00 and 5:00 PM), and in the evening (between 8:00 and 10:00 PM). For the standing test, the subjects laid quietly for 10–30 min, and HR, BP, and ECG were monitored by a multi-lead electrocardiography monitor. A suitable oscillometric BP cuff was used to determine BP. HR was derived automatically from the BP recording. After HR and BP stabilized, subjects were asked to stand upright without support for another 10 min. HR, BP, and ECG were monitored dynamically and recorded at 1, 3, 5, and 10 min after standing (11). The difference between HR at standing 1/3/5/10 min and baseline HR was defined as ΔHR 1 min/ΔHR 3 min/ΔHR 5 min/ΔHR 10 min. The maximum changes in HR, systolic BP (SBP), and diastolic BP (DBP) during the 10-min standing tests compared with those recorded in the supine position were defined as ΔHR_max_, ΔSBP_max_, and ΔDBP_max_, respectively.

### Diagnosis of POTS

The criteria for the diagnosis of POTS were ① associated with frequent OI symptoms, such as dizziness, headache, fatigue, blurred vision, chest tightness, palpitations, tremor in the hands, and even syncope during upright posture; ② associated with a positive HUTT or standing test response: supine HR was normal, and during 10 min of the HUTT or standing test, HR increased ≥40 bpm or the maximum HR ≥130 bpm for children 12 years and younger or ≥125 bpm for adolescents 13 years and older, and there was no significant decrease in BP (SBP decreased <20 mmHg and DBP decreased <10 mmHg); and ③ exclusion of other diseases that could cause OI symptoms, such as cardiovascular, metabolic, neurologic, or autoimmune diseases (1, 11, 13).

### Symptom Score

Symptom score was based primarily on OI symptoms, including dizziness, headache, blurred vision, chest discomfort, palpitations, vomiting, profuse perspiration, and syncope. The frequency of such symptoms was taken into account. Scoring criteria were based on the number of times (on average) a symptom occurred over a particular period: 0 score, the symptom did not occur; 1 score, occurred once per month; 2 scores, 2–4 times per month; 3 scores, 2–7 times per week; and 4 scores, more than once per day. The resulting score was the sum of individual symptom scores (14).

### Statistical Analyses

Data analyses were carried out with the SPSS 25.0 software (IBM Corp, Armonk, NY, USA). Continuous data were expressed as mean ± standard deviation (SD). Data normality was assessed using the Shapiro–Wilk test. Categorical data were expressed as cases (percentage), and the Chi-square test was used for comparisons among groups. The measurement data in multiple groups and hemodynamic parameters of standing tests at different times of the day were compared using one-way ANOVA analysis or the Kruskal–Wallis H test if appropriate. The Bonferroni test was used to adjust for comparisons between the two groups. The receiver operating characteristic (ROC) curve was performed to analyze the sensitivity and specificity of the indicators for predicting the diagnosis. The area under the curve (AUC) represented the predictive values. AUC between 0.5 and 0.7 indicated a relatively low predictive value, between 0.7 and 0.9 had a moderate predictive value, and above 0.9 represented a relatively high predictive value. 95% confidence interval (CI) of the curve area (not including 0.5 or *P* < 0.05) indicated that the index had a value on the prediction of the research result. The cutoff value was determined by the maximum of the Youden index, which was defined as sensitivity plus specificity minus 1. *P* < 0.05 was considered statistically significant.

## Results

### Characteristics of the POTS, Non-POTS, and Control Groups

As shown in Table 1, no statistical difference was found in gender, age, height, weight, and body mass index (BMI) among the POTS, non-POTS, and control groups (*P* > 0.05). Moreover, the median symptom scores were not significantly different between the POTS and non-POTS groups (*P* > 0.05).

**Table 1:** Demographic characteristics and symptom score of the POTS, non-POTS, and control groups.

	**POTS**	**Non-POTS**	**Control**	***P*-value**
Cases, *n*	43	35	26	
Male/female, *n*	24/19	16/19	15/11	0.574
Age, years	12.1 ± 1.7	11.8 ± 1.9	11.7 ± 1.6	0.540
Height, cm	155.3 ± 13.5	151.5 ± 13.5	150.3 ± 12.9	0.268
Weight, kg	41.5 ± 12.1	44.2 ± 13.8	43.3 ± 14.3	0.656
BMI, kg/m^2^	16.8 ± 2.6^a^	18.8 ± 4.0^a^	18.7 ± 3.8^a^	0.05
Symptom score	8.2 ± 4.5^a^	7.5 ± 5.3 ^a^	–	0.372

*Data are mean ± standard deviation or number (percentage). BMI, body mass index*.

a *non-normal distribution*.

### Diurnal Variability in Orthostatic Hemodynamics of the POTS, Non-POTS, and Control Groups

Of the 43 patients with POTS, all 43 patients (100%) met the criterion for POTS hemodynamic response in the morning standing test, 19 patients (44.2%) in the afternoon, and 12 patients (27.9%) in the evening. Among them, only 10 patients (23.3%) met the HR criterion of POTS at all three time points, and another 11 patients (25.6%) met at two time points, including 9 patients in both the morning and afternoon standing tests and 2 patients in both the morning and evening standing tests. Almost half of the POTS patients (51.2%) displayed a positive result only in the morning standing test.

Orthostatic hemodynamic parameters of the three groups were demonstrated in Table 2. At each time point, the baseline HR, SBP, DBP, ΔSBP_max_, and ΔDBP_max_ were not significantly different among the POTS, non-POTS, and control groups (*P* > 0.05). However, after the Bonferroni test, the orthostatic parameters of HR at the three time points, including ΔHR and absolute standing HR from 1 to 10 min after standing and ΔHR_max_, were the highest in the POTS group compared with in the non-POTS and control groups (*P* < 0.05) except for the afternoon standing HR at 1 min (*P* > 0.05). The morning ΔHR_max_, ΔHR 3 min, ΔHR 5 min, and ΔHR 10 min in the non-POTS group were significantly higher than those in the control group (*P* < 0.05). Nevertheless, there was no significant difference in orthostatic hemodynamic parameters in the afternoon and evening tests between the non-POTS and control groups (*P* > 0.05).

**Table 2:** Orthostatic hemodynamic characteristics of the POTS, non-POTS, and control groups.

**Items**	**POTS**	**Non-POTS**	**Control**	***P*-value**
**Morning standing test**				
Baseline HR, bpm	72.5 ± 10.6	72.2 ± 7.4^****^†^****^	76.4 ± 10.0	0.172
Standing HR 1 min, bpm	111.2 ± 13.5^^*^^#^^	99.7 ± 10.6	98.1 ± 12.7	<0.001
Standing HR 3 min, bpm	115.9 ± 14.3^^*^^#^^	98.8 ± 8.4^^#^^	95.4 ± 10.7	<0.001
Standing HR 5 min, bpm	118.0 ± 14.9^*^^^†^^#^^	99.7 ± 7.5^^#^^	97.7 ± 10.2	<0.001
Standing HR 10 min, bpm	118.5 ± 12.5^^*^^†^^#^^	101.5 ± 9.4	99.7 ± 10.0	<0.001
ΔHR_max_, bpm	49.0 ± 7.8^^*^^†^^#^^	30.5 ± 5.7^^*^^†^^#^^	24.4 ± 7.1	<0.001
ΔHR 1 min, bpm	39.4 ± 11.5^^*^^†^^#^^	27.4 ± 8.2^**^#^**^	21.7 ± 8.6	<0.001
ΔHR 3 min, bpm	43.8 ± 11.1^^*^^†^^#^^	26.6 ± 7.7^^*^^†^^#^^	19.0 ± 9.5	<0.001
ΔHR 5 min, bpm	45.50 ± 11.0^^*^^†^^#^^	27.4 ± 6.0^^*^^†^^#^^	21.2 ± 6.7	<0.001
ΔHR 10 min, bpm	45.8 ± 6.7^^*^^†^^#^^	29.3 ± 6.3^^*^^†^^#^^	23.3 ± 7.3	<0.001
Baseline SBP, mmHg	105.1 ± 8.3	105.5 ± 8.5	109.1 ± 8.6	0.148
Baseline DBP, mmHg	57.3 ± 8.2	57.9 ± 6.8	57.9 ± 5.8	0.910
ΔSBP_max_, mmHg	5.3 ± 10.6^a^	5.5 ± 8.1^a^	5.8 ± 9.8^a^	0.994
ΔDBP_max_, mmHg	11.8 ± 8.8	11.9 ± 8.6	11.4 ± 5.9	0.884
**Afternoon standing test**				
Baseline HR, bpm	74.7 ± 9.8	77.9 ± 10.4	75.9 ± 9.6	0.383
Standing HR 1 min, bpm	104.8 ± 12.2	100.9 ± 13.3	98.4 ± 13.4	0.130
Standing HR 3 min, bpm	108.6 ± 13.4^*^	97.3 ± 14.6	96.2 ± 9.7	<0.001
Standing HR 5 min, bpm	109.1 ± 13.6^*^	98.2 ± 12.8	96.4 ± 8.7	<0.001
Standing HR 10 min, bpm	110.1 ± 14.7^*^	99.0 ± 15.4	98.4 ± 8.0	<0.001
ΔHR_max_, bpm	38.0 ± 11.9^*^	23.3 ± 9.7	22.5 ± 10.1	<0.001
ΔHR 1 min, bpm	30.1 ± 10.6^*^	23.0 ± 10.6	22.4 ± 10.3	0.003
ΔHR 3 min, bpm	33.9 ± 10.8^^*^^#^^	19.3 ± 11.0	20.3 ± 7.8	<0.001
ΔHR 5 min, bpm	34.4 ± 10.5^*^	20.3 ± 8.9	20.5 ± 7.0	<0.001
ΔHR 10 min, bpm	35.8 ± 13.2^*^	21.7 ± 8.6	22.8 ± 8.1	<0.001
Baseline SBP, mmHg	104.4 ± 10.0	105.1 ± 8.9	107.4 ± 6.9	0.402
Baseline DBP, mmHg	57.4 ± 5.9	56.6 ± 6.9	57.1 ± 6.7	0.869
ΔSBP_max_, mmHg	6.4 ± 9.5^a^	6.3 ± 7.6^a^	7.0 ± 5.9 ^a^	0.878
ΔDBP_max_, mmHg	10.9 ± 6.0	10.7 ± 5.0	9.6 ± 7.8	0.702
**Evening standing test**				
Baseline HR, bpm	74.7 ± 11.7	73.6 ± 8.4	74.0 ± 10.0	0.887
Standing HR 1 min, bpm	101.6 ± 15.3^*^	94.1 ± 10.0	92.8 ± 10.2	0.009
Standing HR 3 min, bpm	102.5 ± 14.8^*^	90.9 ± 9.9	92.5 ± 9.8	<0.001
Standing HR 5 min, bpm	103.6 ± 15.8^*^	92.1 ± 10.2	94.0 ± 8.0	<0.001
Standing HR 10 min, bpm	106.8 ± 13.8^*^	94.6 ± 11.7	93.9 ± 8.6	<0.001
ΔHR_max_, bpm	33.0 ± 11.6^*^	22.9 ± 7.6	22.0 ± 9.8	<0.001
ΔHR 1 min, bpm	26.9 ± 10^*^	20.2 ± 8.0	18.9 ± 10.4	0.002
ΔHR 3 min, bpm	27.8 ± 10.0^*^	17.4 ± 8.4	18.5 ± 9.3	<0.001
ΔHR 5 min, bpm	28.9 ± 101.0^*^	18.5 ± 8.1	20.0 ± 8.8	<0.001
ΔHR 10 min, bpm	31.4 ± 11.7^*^	20.9 ± 8.6	19.9 ± 10.2	<0.001
Baseline SBP, mmHg	103.5 ± 7.7	105.2 ± 8.2	106.7 ± 8.1	0.299
Baseline DBP, mmHg	55.0 ± 6.2	55.4 ± 4.9	57.3 ± 6.1	0.310
ΔSBP_max_, mmHg	5.5 ± 9.5^a^	5.3 ± 6.4^a^	4.4 ± 7.9^a^	0.562
ΔDBP_max_, mmHg	14.0 ± 5.5	13.9 ± 7.4	11.1 ± 5.2	0.153

*Data are mean ± standard deviation*.

a *non-normal distribution. HR, heart rate; SBP, systolic blood pressure; DBP, diastolic blood pressure; ΔHR 1/3/5/10 min, heart rate change from supine to standing position at 1, 3, 5, and 10 min; ΔHR/SBP/DBP_max_, the maximum change of HR/SBP/DBP during the 10 min of standing test. After the Bonferroni test*,

* *significant difference from other groups (P < 0.05);*

† *significant difference compared with that measured in the afternoon test (P < 0.05);*

# *significant difference compared with that measured in the evening test (P < 0.05)*.

**Figure 1:**
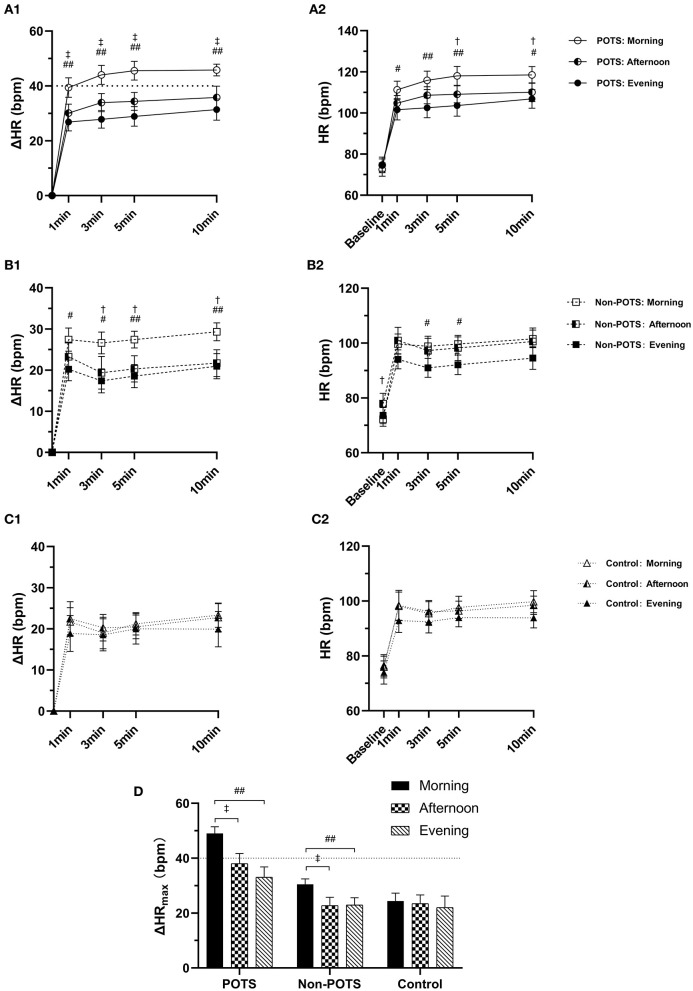
Diurnal variability in orthostatic parameters of HR among the POTS, non-POTS, and control groups. **(A1, B1, C1)** The heart rate change from supine to standing (ΔHR) at 1, 3, 5, and 10 min in the POTS, non-POTS, and control groups, respectively. **(A2, B2, C2)** Absolute HR at baseline and 1, 3, 5, and 10 min after standing in the POTS, non-POTS, and control groups, respectively. **(D)** The maximum HR increment (ΔHRmax) during the 10-min standing test in the POTS, non-POTS, and control groups. Data are mean ± 95% CI. After Bonferroni test, ^†^*P* < 0.05, ‡ *P* < 0.001 significant difference compared with that measured in the afternoon test.# *P* < 0.05, ## *P* < 0.001 significant difference compared with that measured in the evening test (*P* < 0.05).

**Figure 2:**
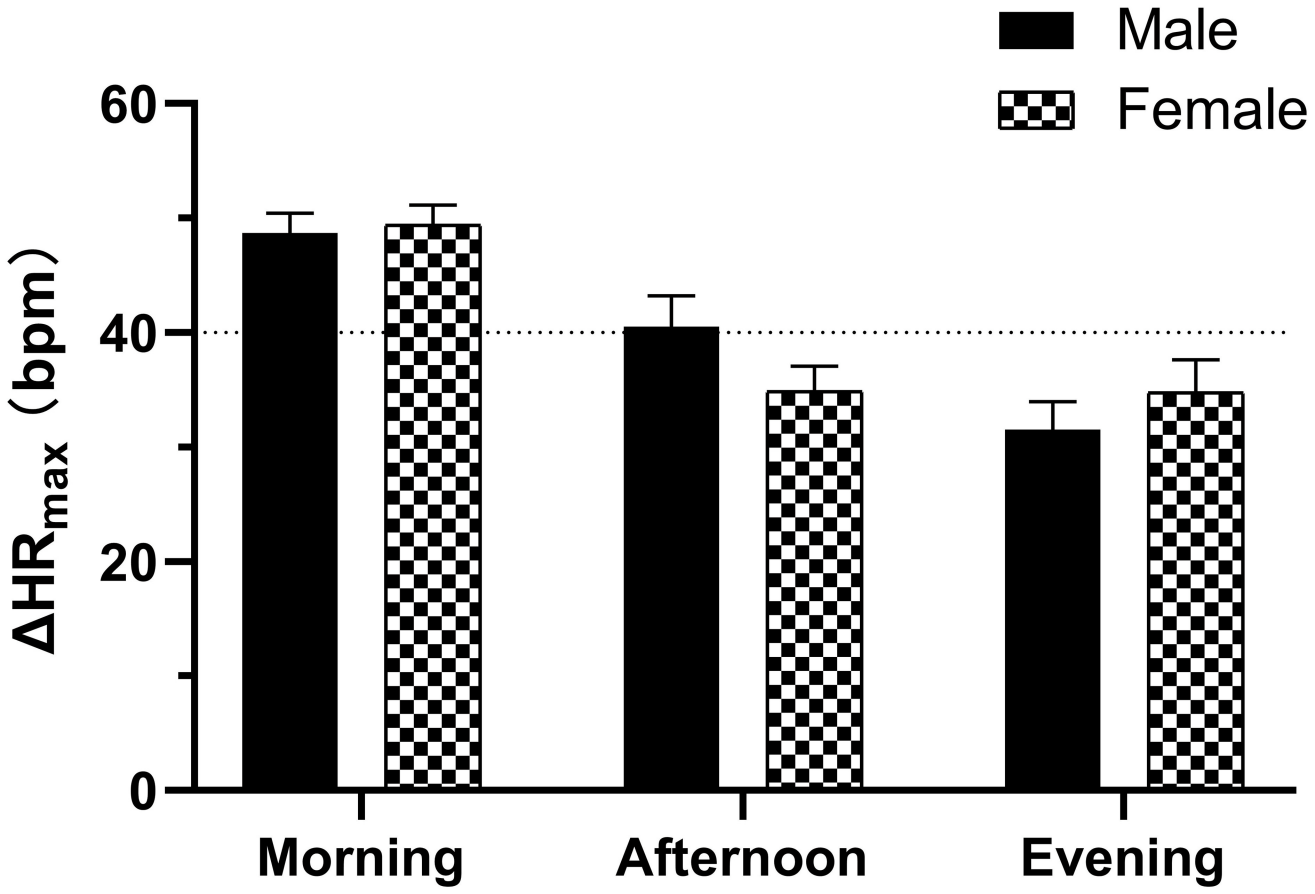
Diurnal variability of the maximum HR increment (ΔHRmax) in the standing tests between males and females with POTS. Data are mean ± 95% CI.

Of all subjects, the baseline HR, SBP and DBP, ΔSBP_max_, and ΔDBP_max_ did not differ with the time of day (*P* > 0.05) except for the baseline HR in the non-POTS group (*P* < 0.05). In the healthy control group, the orthostatic parameters of HR were not significantly different with the time of day (*P* > 0.05). In contrast, the orthostatic parameters of HR showed diurnal variability in both the POTS and non-POTS groups. These parameters were significantly greater in the morning than in the afternoon and/or in the evening tests (*P* < 0.05). However, these parameters were not significantly different between the afternoon and evening tests (*P* > 0.05) (Figure 1). Additionally, there was no significant difference in ΔHR_max_ at the three time points between males and females for the POTS group (*P* > 0.05) (Figure 2).

According to ΔHR_max_ during the 10-min standing test, then, the subjects were divided into three subtypes: those with ΔHR_max_ <30 bpm, those with ΔHR_max_ 30–39 bpm, and those with ΔHR_max_ ≥40 bpm. The distribution in subtypes of ΔHR_max_ among the POTS, non-POTS, and healthy control groups was seen in Table 3. We used the Chi-square test to compare the percentage of subjects with ΔHR_max_ <30 bpm and with ΔHR_max_ 30–39 bpm between the non-POTS and control groups. In the morning test, the percentage of subjects with ΔHR_max_ 30–39 bpm in the non-POTS group was significantly higher than that in the control group (57.1 vs. 23.1%, *P* = 0.008). Nevertheless, there was no significant difference between the two groups in the afternoon and evening tests (*P* > 0.05).

**Table 3:** The distribution in subtypes of ΔHRmax among the POTS, non-POTS, and control groups.

		**POTS**	**Non-POTS**	**Control**	***P*-value**
**Morning standing test**	ΔHR_max_ ≥ 40 bpm	43 (100%)	0	0	
	ΔHR_max_ 30–39 bpm	0	20 (57.1%)^*^	6 (23.1%)	0.008
	ΔHR_max_ < 30 bpm	0	15 (42.9%)^*^	19 (76.9%)	
**Afternoon standing test**	ΔHR_max_ ≥ 40 bpm	19 (44.2%)	0	0	
	ΔHR_max_ 30–39 bpm	17 (39.5%)^*^	10 (28.6%)	7 (26.9%)	0.001
	ΔHR_max_ < 30 bpm	7 (16.3%)^*^	25 (71.4%)	19 (73.1%)	
**Evening standing test**	ΔHR_max_ ≥ 40 bpm	12 (27.9%)	0	0	
	ΔHR_max_ 30–39 bpm	13 (30.2%)	8 (22.9%)	8 (30.8%)	0.249
	ΔHR_max_ < 30 bpm	18 (41.9%)	27 (77.1%)	18 (69.2%)	

*ΔHR_max_, the maximum change of HR during the 10 min of standing test*.

* *Significant difference from other groups (P < 0.05)*.

### ROC Curve Analysis of the Orthostatic HR Parameters at Different Time Points for the Prediction of the POTS Diagnosis

**Figure 3:**
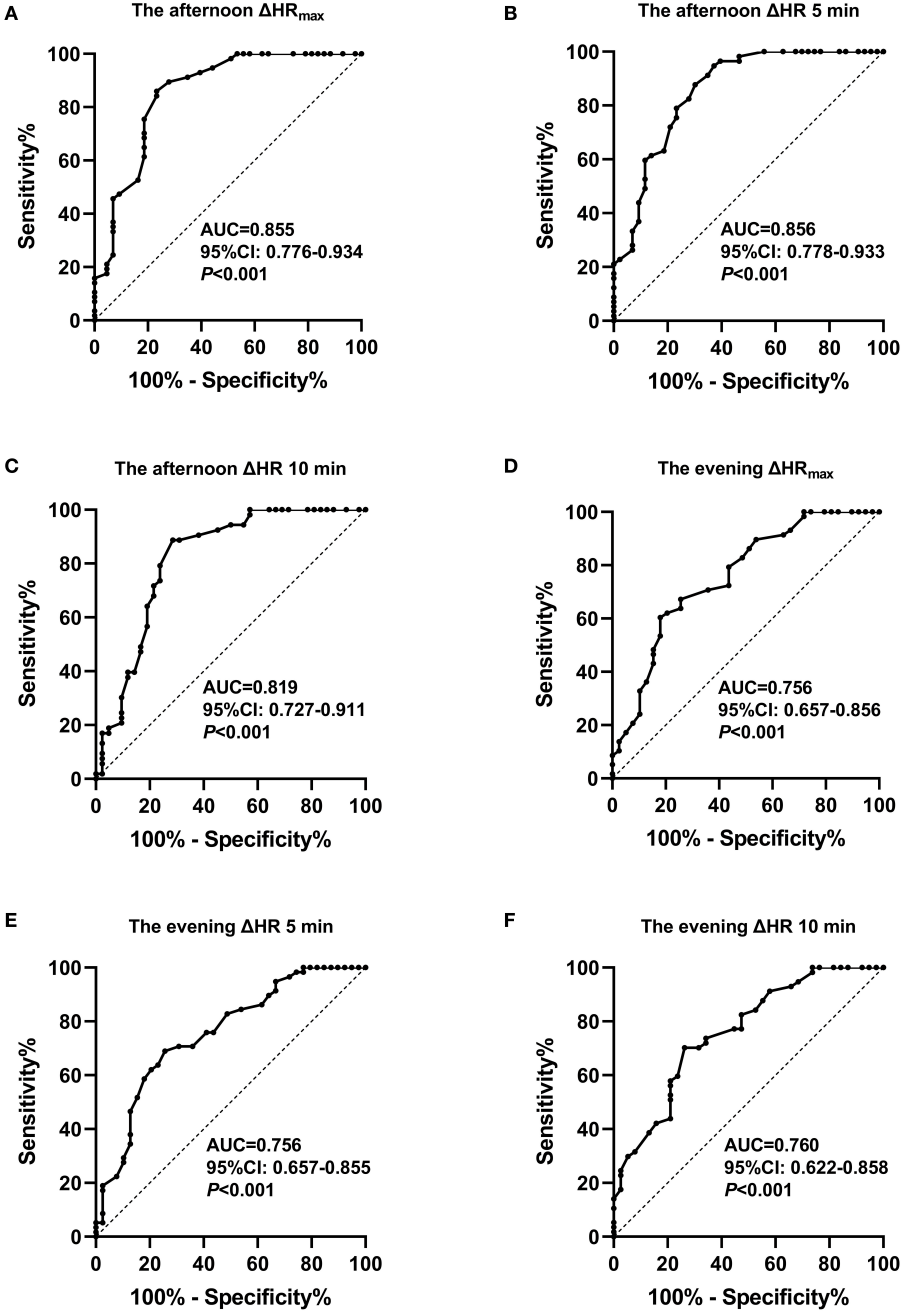
Receiver operating characteristic curve analysis of orthostatic heart rate parameters in the afternoon and evening standing tests to predict the POTS diagnosis. **(A)** The maximum heart rate increment (ΔHR_max_) in the afternoon standing test. **(B)** ΔHR at 5 min in the afternoon standing test. **(C)** ΔHR at 10 min in the afternoon standing test. **(D)** ΔHR_max_ in the evening standing test. **(E)** ΔHR at 5 min in the evening standing test. **(F)** ΔHR at 10 min in the evening standing test.

The ROC curve was used to analyze ΔHR at different time points to predict the POTS diagnosis (Figure 3). The AUC values for ΔHR_max_, ΔHR 5 min, and ΔHR 10 min in the afternoon standing test were 0.855 (95% CI 0.776–0.934), 0.856 (95% CI 0.778–0.933), and 0.819 (95% CI 0.727–0.911), respectively, for the prediction of the POTS diagnosis. Cutoff values for ΔHR_max_, ΔHR 5 min, and ΔHR 10 min were 32, 32, and 31 bpm, yielding sensitivities of 76.7, 62.8, and 71.4% and specificities of 86, 94.7, and 88.7%, respectively. The AUC values for ΔHR_max_, ΔHR 5 min, and ΔHR 10 min in the evening standing test were 0.756 (95% CI 0.657–0.856), 0.756 (95% CI 0.657–0.855), and 0.760 (95% CI 0.622–0.858), respectively, for the prediction of the POTS diagnosis. Cutoff values for ΔHR_max_, ΔHR 5 min, and ΔHR 10 min were 25, 23, and 25 bpm, yielding sensitivities of 82.1, 74.4, and 73.7% and specificities of 60.3, 69, and 70.2%, respectively. For easy memory, we suggest that orthostatic HR increment ≥30 bpm in the afternoon standing test and orthostatic HR increment ≥25 bpm in the evening standing test could be considered as POTS, combined with clinical symptoms.

The external validation test in the predictive values of the afternoon and evening ΔHR_max_ was shown in Table 4. At the afternoon ΔHR_max_ ≥30 bpm, its sensitivity, specificity, and accuracy in the POTS diagnosis were 85, 71.4, and 78%, respectively. Furthermore, at the evening ΔHR_max_ ≥25 bpm, its sensitivity, specificity, and accuracy in the POTS diagnosis by the evening ΔHR_max_ were 85.0, 76.2, and 80.5%, respectively.

**Table 4:** The predictive values of the afternoon and evening ΔHR_max_ in the external validation test.

**Items**	**Cutoff value**	**Clinical diagnosis**, ***n*** **(%)**	
		**POTS (*n* = 20)**	**Non-POTS (*n* = 21)**
Afternoon standing test	ΔHR_max_ ≥ 30 bpm	17 (85%)	6 (28.6%)
	ΔHR_max_ < 30 bpm	3 (15%)	15 (71.4%)
Evening standing test	ΔHR_max_ ≥ 25 bpm	17 (85%)	5 (23.8%)
	ΔHR_max_ < 25 bpm	3 (15%)	16 (76.2%)

*ΔHR_max_, the maximum change of HR during the 10 min of standing test*.

## Discussion

In this study, we showed that the orthostatic HR increment exhibited diurnal variability in children and adolescents with OI, with a peak in the morning. In contrast, the orthostatic hemodynamics did not change with the time of day in the healthy control group. The orthostatic HR increment in the non-POTS group was higher only in the morning standing test than that in the control group. However, the orthostatic HR increment in the POTS group was markedly the highest in the whole day compared with that in the non-POTS and control groups. Despite the higher HR increment, not all POTS patients can meet the HR criterion of a 40-bpm threshold throughout the day. Only half of the POTS patients (51.2%) displayed a positive result only in the morning standing test. Next, we used the ROC curve analysis to explore the predictive values of orthostatic HR parameters in the afternoon and evening standing tests for the POTS diagnosis. The ΔHR_max_ and ΔHR at 5 and 10 min in the afternoon and evening tests showed moderate predictive values. On our validation test, with 30 bpm as the cutoff value of the afternoon ΔHR_max_, its sensitivity, specificity, and accuracy in the POTS diagnosis were 85, 71.4, and 78%, respectively. Moreover, with 25 bpm as the cutoff value of the evening ΔHR_max_, its sensitivity, specificity, and accuracy in the POTS diagnosis were 85.0, 76.2, and 80.5%, respectively.

The diagnosis of POTS is based on the clinical history and the excessive increment in HR after standing without a specific biomarker. The active standing test and the passive HUTT are two main and comparable methods for the assessment of POTS (15). We used the two methods for the POTS diagnosis to exclude transient orthostatic tachycardia. Our study revealed that the morning standing test showed a higher positive rate for the POTS diagnosis than the afternoon and evening standing tests. It implies that the time of day can affect the diagnostic accuracy of the standing test for POTS. Ignoring the diurnal variability of orthostatic HR increment could result in a missed diagnosis of POTS. In our study, although all POTS patients met the HR criterion with ≥40 bpm in the morning standing test, the positive diagnostic rate decreased to 44.2% in the afternoon and 27.9% in the evening. Despite the relatively low positive rate in the afternoon and evening, the orthostatic HR increment in the POTS group was still markedly larger than that in the non-POTS and control groups. We suggest either implementing a standing test in the early morning or redefining the hemodynamic criteria based on diurnal variability. Our findings indicate that a cutoff value of 30 bpm for the afternoon ΔHR_max_ and a cutoff value of 25 bpm for the evening ΔHR_max_ to diagnose POTS yielded relatively favorable sensitivity and specificity, which were confirmed by external validation test.

Boris et al. (12) reported that the burden of OI symptoms was unrelated to the degree of tachycardiac response to an upright position. In our study, the POTS (HR increased ≥40 bpm) and non-POTS (HR increased <40 bpm) patients had similar symptom scores. Although the non-POTS patients had significantly larger HR increment than the healthy controls in the morning standing test, the above difference was diminished in the afternoon and evening tests between the non-POTS and control groups.

Another key finding in our study is the diurnal variability of orthostatic HR increment in children and adolescents with POTS. The most probable explanation for the exaggerated orthostatic HR increment observed in the morning is the intrinsic circadian variability. Increasing or decreasing HR is modulated *via* two opposite pathways of autonomic nervous systems—the catecholaminergic and sympathetic nervous systems and the acetylcholinergic and parasympathetic nervous systems. Plasma catecholamines epinephrine (an indicator of adrenal medullary activity) and norepinephrine (an indicator of sympathetic nervous system activity) levels show significant diurnal variability, with rapid increase after waking and a peak in the late morning, then a gradual decrease throughout the day (16, 17). Plasma norepinephrine levels increase sharply from supine to upright posture, but epinephrine levels are less affected, suggesting that the former is mainly influenced by posture (16, 18). Up to 50% of the POTS patients exhibit a hyperadrenergic state with standing plasma norepinephrine ≥600 pg/ml (13). Salivary cortisol levels can reflect plasma cortisol levels and are positively correlated with norepinephrine levels. Lin et al. (19) reported that salivary cortisol levels were significantly higher in the POTS children than in the control children at all examined time points, with peaking shortly after waking and decreasing during the night. Furthermore, our previous study reported that the rate–pressure product values (multiplying HR and SBP) of the POTS children were significantly higher than those of the controls in the early morning, paralleled with the morning surge of orthostatic HR increment (20).

POTS patients have impaired α1 adrenergic receptor (α1 AR)-mediated vasoconstriction and enhanced β1 adrenergic receptor (β1 AR)-mediated tachycardia (21, 22). Several studies have investigated the presence of autoantibodies in POTS patients, and these autoantibodies induced an allosterically mediated positive modulatory effect on β1 AR and negative modulatory effect on α1 AR activity (23, 24). The cardiac output shows diurnal variability with a steep increase in the early morning, whereas the total peripheral resistance shows an unexpected decrease in the morning (25). Panza et al. (26) showed that the vasodilator effect of phentolamine (an α AR-antagonist drug) was significantly greater in the early morning than in the afternoon and evening. Therefore, when a POTS patient stands from a supine position, α1 AR-mediated vasoconstriction to upright posture could be blunter in the early morning, leading to a further increase of norepinephrine release. Some POTS patients are hypersensitive to β1 AR. The positive chronotropic effect of β1 AR would be enhanced in the morning. Another acknowledged mechanism is central hypovolemia (1, 2). The morning standing test was performed after an overnight fast and overnight bed rest. The hypovolemic state may be exacerbated in the morning resulting from the overnight fast and morning urination.

The exaggerated response to adrenergic receptors, the increased catecholamine levels, and the exacerbated hypovolemic state in the morning may explain the excessive HR increment after standing observed in the morning for POTS children and adolescents.

Despite the promising results, our study still had limitations. This was a single-center study, and the number of cases was not large enough. In addition, all the participants in our study kept their usual routine of daily activities on the day of examination. The diurnal variability of HR is influenced by the physical activities, which needs the participants to avoid strenuous exercise and emotional excitement. More studies with larger sample-sized multicenter studies are needed in the future to establish and validate the hemodynamic criteria for POTS based on diurnal variability.

## Conclusions

In summary, we emphasize the significance of diurnal variability for the POTS diagnosis. The diagnostic test performed in the early morning would be the most sensitive for POTS hemodynamic positivity. Nevertheless, new diagnostic criteria for POTS combined with the concept of diurnal variability should be established shortly, which makes the diagnostic test free from the restriction of the time of day.

## Data Availability Statement

The data analyzed in this study is subject to the following licenses/restrictions: The datasets generated for this study are available on request to the corresponding author. Requests to access these datasets should be directed to Cheng Wang, wangcheng2nd@csu.edu.cn.

## Ethics Statement

The studies involving human participants were reviewed and approved by the Medical Ethical Committee, The Second Xiangya Hospital, Central South University review board approved the study, with waiver of requirement for informed consent. Written informed consent to participate in this study was provided by the participants' legal guardian/next of kin.

## Author Contributions

HC and CW conceived the study. SW, RZ, FL, JZ, YW, and YX collected and reviewed the subjects' data. SW and RZ performed the statistical analysis. HC drafted the manuscript and all authors contributed to its revision. All authors contributed to the article and approved the submitted version.

## Conflict of Interest

The authors declare that the research was conducted in the absence of any commercial or financial relationships that could be construed as a potential conflict of interest.
